# A comparison of the effects of three different estrogen used for endometrium preparation on the outcome of day 5 frozen embryo transfer cycle

**DOI:** 10.5935/1518-0557.20200059

**Published:** 2021

**Authors:** Juliano Brum Scheffer, Bruno Brum Scheffer, Ana Paula de Souza Aguiar, Juliana Baumgratz Franca, Daniel Mendez Lozano, Renato Fanchin

**Affiliations:** 1 IBRRA - Brazilian Institute of Assisted Reproduction, Belo Horizonte, Brazil; 2 School of Medicine, Tecnologico de Monterrey and Center for Reproductive Medicine CREASIS, San Pedro Monterrey, Mexico; 3 Professeur des Universites - Praticien Hospitalier en Medecine de la Reproduction, France; Hopital Foch, France

**Keywords:** endometrium preparation, frozen embryo, thawed embryo, estradiol, estrogen, pregnancy rate

## Abstract

**Objective:**

To evaluate the effects of three different estrogen used for endometrium preparation on pregnancy rate, as well as hormone profile on day 5 frozen embryo transfer (FET) cycles.

**Methods:**

Retrospective, observational study. Setting: A tertiary teaching and research private reproductive medicine center. Patients: Ninety patients who were undergoing endometrium preparation for day five frozen embryo transfer cycle (FET). Intervention(s): The women were divided in three groups according to the administration route of estrogen (E2): oral (Primogyna), transdermal patches (Estradot), or transdermal gel (Oestrogel Pump). These administration routines of estrogen are equivalent to 6mg of estradiol daily. All women received 600mg of vaginal progesterone (P) per day (Utrogestan) for luteal phase support. We drew blood samples on starting P day, as well as on beta hCG day for E2 and P measurements. Main Outcome Measure(s): Clinical pregnancy rates (PR).

**Results:**

Patient features in the three groups were comparable. There were no significant differences concerning implantation rate, clinical PR, miscarriage rate, multiple-pregnancy rate, or E2 and P levels on starting P day and on beta hCG day.

**Conclusions:**

In FET cycles with oral (Primogyna) or transdermal patches (Estradot), or transdermal gel (Oestrogel Pump), there was no significant difference on pregnancy rates.

## INTRODUCTION

Embryo implantation is the successful invasion of the endometrium by the blastocyst. The endometrium, which undergoes a series of structural and biochemical changes during the reproductive cycle, must also be in a receptive phase, because a normal, healthy endometrium will resist implantation in all other phases of the cycles.

In frozen embryo transfer (FET) cycles, estrogen and progesterone are sequentially administered to synchronize the embryo transfer with the endometrial window of implantation. The increased use of FET has allowed investigators to closely examine various aspects of this treatment strategy (Michalas *et al*., 1996; Borini *et al*., 2001).

There is, in current usage, a wide variety of regimens that can be used to attain endometrial receptivity. Varying doses and routes of administration are available for both estrogen (E2) and progesterone (P). One systematic review and three Cochrane Reviews, from 2008 at 2017, concluded that there is “insufficient evidence to recommend one particular protocol for endometrial preparation over another about pregnancy rates after embryo transfers (Ghobara & Vandekerchove, 2008; Glujovsky *et al*., 2010; Ghobara *et al*., 2017; Groenewoud *et al*., 2013).

The purpose of E2 priming and attainment of endometrial proliferation is the induction of P receptors, which allows subsequent P stimulation to induce endometrial receptivity. Nevertheless, one must keep in mind that estrogenic stimulation may have a significant effect on the subsequent luteal phase, and the luteal progression of the endometrium depends not only on the duration and strength of P stimulation, but also on the prior E2 priming. Therefore, an apparent endometrial delay in response to P may be reflective of insufficient P receptors resulting from inadequate E2 priming (Lessey *et al.,* 1988).

With an adequate estrogen regimen, the endometrium and, more precisely, its P receptors, are primed and ready to induce a receptive environment for the developing embryo. Estrogen is continued as daily progesterone administration is initiated 5 days before the scheduled embryo transfer. Upon the addition of P, the endometrium undergoes both conformational and biochemical changes to produce an environment capable of supporting embryo implantation (Kodaman & Taylor, 2004). 

Through this study, we aimed to evaluate whether the dosage and mode of administration of estrogen on endometrium preparation for FET interferes with the levels of estrogen on thawing embryo day and beta hCG day and pregnancy rate. 

## MATERIALS AND METHODS

### Patients

Our retrospective study included 90 women undergoing endometrium preparation for day five frozen embryo transfer cycle at the private reproductive medicine center Brazilian Institute of Assisted Reproduction - (IBRRA) between April 2018 and February 2019.

The inclusion criteria were: i) patients using frozen blastocysts derived from previous stimulation cycles, ii) no current or past diseases affecting ovaries or gonadotropin, or sex steroid secretion, clearance, or excretion, iii) no current hormone therapy, iv) adequate visualization of ovaries by transvaginal ultrasound, v) two or more frozen type A and/or B embryos on day 5 and vi) patients with an endometrial thickness >7mm by 10-14 days from initiating estrogen supplementation. All the patients signed the informed consent form. The investigation was approved by the Institutional Review Board and by the IBRRA Ethics Committee (Protocol number 22435/2019).

### Treatment Protocol

All the patients underwent suppression of their hypothalamic-pituitary-ovarian axis with oral contraceptive pills for 21 days. After the cessation of oral contraceptives for 4 days, the patients had formal evaluation of their endometrial cavity via three dimensional transvaginal ultrasonography scans (TVUS) and assessment of serum estrogen, progesterone and luteinizing hormone to confirm that they were in the early proliferative phase of their menstrual cycles, and to rule out pregnancy.

For endometrium preparation, we assigned the patients into three groups of 30 patients: transdermal estrogen gel daily (Oestrogel pump, Estradiol- Besins Pharmaceuticals, Belgium - Group 1), oral estrogen daily (Primogyna - estradiol valerate, Bayer Pharmaceuticals, Germany - Group 2), or transdermal estrogen patches daily (Estradot- Estradiol, Novartis Pharmaceuticals, Swiss - Group 3). The three different administration routines of estrogen were each equivalent to 6 mg of estradiol daily. We performed TVUS every week to assess the recipient endometrium, with the first ultrasound occurring within 7 a 10 days of initiating estrogen supplementation. We measured serum progesterone at each visit to rule out premature ovulation, before staring progesterone supplementation.

Once the FET timing was determined, all patients began supplementation with 600mg once daily with intravaginal P (Utrogestan, progesterone micronized, Besins Pharmaceuticals, Belgium). On the fifth day of progesterone administration, we selected a vitrified blastocyst for transfer based on graded blastocyst (A and/or B) by Gardner’s grading scale (Gardner & Schoolcraft, 1999). 

The same embryologist performed all embryology and embryo scoring in this study. All women received 1 or 2 embryos classified as A and/or B. The embryos were thawed on the day of transfer (blastocyst) in Sydney IVF Blastocyst Medium (Cook Medical, Canada). We determined the number of embryos transferred by following the Federal Board of Medicine - Brazil (FCM) guidelines. Other authors described the vitrification and thawing procedure (Kuwayama *et al*., 2005).

We drew other blood samples on the day of thawing the embryo and on beta hCG day (2 weeks after ET) for E2 and P measurements. We continued with the estrogen administration and intravaginal P until pregnancy was ruled out by a negative serum beta-hCG measurement performed on day 14 after ET, and until the 12^th^ week of pregnancy for pregnant patients. We confirmed clinical pregnancies with the confirmation of positive fetal cardiac activities by transvaginal sonography. There were no drug-related side effects. 

We transferred the embryos 5 days after starting the progesterone. We instructed the to have a full bladder, which would provide an acoustic window for visualizing the uterus, in preparation for the ultrasound-guided embryo transfer. We placed the patients in the dorsal lithotomy position without anesthesia or sedation. We performed each embryo transfer with a Wallace Classic Soft Embryo Transfer Catheter, and performed an abdominal ultrasound using a 5 MHz probe (GE Logiq 400 Pro Series, General Electric Company, Pewaukee, WI). 

### Laboratory Methods

We determined E2 and P levels by electrochemiluminescence immunoassay (Elecsys and Cobas e analyzers; Roche Diagnostics GmbH, Mannheim, Germany). We established the results via a calibration curve specifically generated for the instrument by a two-point calibration and a provided master curve. The sensitivity analysis was 5 pg/mL, and the linear interval of the test was 5 to 4,300 pg/mL for estrogen. E2 levels were determined with intra-assay and interassay coefficients of variation, of <3.3% and <4.9%, respectively. Sensitivity analysis was 0.21 ng/mL, and the linear interval of the test was 0.21 to 60 ng/mL for P. P levels were assayed with intra-assay and interassay coefficients of variation of <8% and <9.1%, respectively. 

### Statistical Analysis

We assessed the data using the SPSS for Windows, release 15.0 (SPSS, Inc., Chicago, IL). We expressed the continuous data as means ± SD and analyzed with one-way ANOVA tests for normally distributed data and with the Kruskal-Wallis test for other data. We used the Pearson’s c^2^ test to analyze the categorical data. Upon finding statistical differences, the groups were compared by using the c^2^ test with Spearman correction. We analyzed the E2, P, and E2/P rates for ongoing pregnancies in all groups using the Mann-Whitney U test. The significance threshold was 5%. 

## RESULTS

### Patient characteristics

Our retrospective study included 90 patients. Group 1 was composed of 30 patients, Group 2 of 30 patients, and Group 3 of 30 patients.

Table 1 described the patient’s characteristics. There was no significantly different pattern between the three groups concerning age, body mass index (BMI), day 3 FSH and day 3 E_2_, E_2_ p level on thawing embryo day and on beta hCG day and P level on thawing embryo day and on beta hCG day.

**Table 1:** Patient and cycle characteristics for the three treatment groups

Characteristic	Group 1 (E2 gel) n=30	Group 2 (E2 oral) n=30	Group 3 (E2 patch) n=30	*p* value
**Age (y)**	**35.61±2.34**	35.5±3.81	34.81±3.49	.70
BMI (Kg/m^2^)	23.48±2.12	24.23±4.80	24.32±2.10	.17
Day 3 FSH(mUI/mL	11.35±5.40	12.00±6.73	15.71±6.41	.42
Length of preparation of only estrogen (d)	15.00±0.66	14.75±1.92	15.61±1.91	.13
Peak E2 level on thawing day embryo(pg/ml)	301.00±73.97	298.00±55.49	302.64±57.50	.16
Peak E2 level on beta day (pg/ml)	398.62±63.97	385.00±74.49	379.64±67.50	.12
Peak P level on thawing day embryo(ng/ml)	0.27±0.12	0.28±0.16	0.27±0.23	.14
Peak P level on beta day (ng/ml)	0.35±0.22	0.38±0.26	0.40±0.23	.09

Note: *p*< .05 was considered statistically significant. Data is expressed as mean ± SD

### ART Outcome

There was no significant difference in the number of thawed embryos A + B, the number of embryos transferred, implantation rates, clinical PR, miscarriage rates, multiple-pregnancy rates (Table 2).

**Table 2:** Frozen embryo transfer cycle characteristics of the three treatment groups

Characteristic	Group 1 (E2 gel) n=30	Group 2 (E2 oral) n=30	Group 3 (E2 patch) n=30	*p* value
Nº of embryos thawed A and/or B	1.66±0.41	1.67±0.48	1.12±0.85	.38
Nº of embryos transferred	1.76±0.17	1.80±0.29	1.90±0.30	.54
Implantation rate (%)	22.32±27.99	21.78±30.85	19.94±20.87	.79
Clinical PR, % (nº)	46.6 (14/30)	46.6 (14/30)	50.0 (15/30)	.84
Miscarriage rate % (nº)	14.2 (2/14)	14.2 (2/14)	13.3 (2/15)	.43
Multiple-pregnancy rate, % (nº)	3.33 (1/30)	3.33 (1/30)	3.33 (1/30)	.65
Δ E_2_level on thawing day/beta day	88.28±4.53	80.93±4.14	79.41±5.38	.13
Δ P level on thawing day/beta day	0.12±0.13	0.12±0.14	0.11±0.18	.15

Note: *p*< .05 was considered statistically significant. Data is expressed as mean ± SD or as percentage and number. Δ - Mean of Variation of hormone profile

### Hormonal profile

There was no significant difference in the variance rate between E2 on thawing embryo day and beta HCG in the three groups (*p*>0.05). The E2/P ratio on the beta HCG day was comparable between the three groups, demonstrating no different effect on the hormonal profile regarding the mode of estrogen administration (Table 3).

**Table 3:** Comparison of hormone profile variance rate for the three treatment groups

Variance rate of hormone profile	*p* value		
	Group 1 x 2	Group 1 x 3	Group 2 x 3
E_2_thawed day/E_2_beta hCG day	.09	.06	.06
E_2_beta hCG day/ P hCG day	.08	.08	0.9

*Note: p*< .05 was considered statistically significant

### Clinical Pregnancy

In relation to the hormonal profile, a positive test of pregnancy was significantly associated with the E2 level on the beta hCG day (r=0.73 *p*<.0001), independently of estrogen protocols.

Thus, the mode of administration of estrogen did not interfere with any ART outcome.

## DISCUSSION

Supraphysiological estrogen levels alter the expression of genes and implantation factors in the perimplantation endometrium (Chang *et al*., 2011). Interestingly, despite the elevated circulating serum estrogen levels associated with artificial endometrial preparation before FET, studies have failed to show that undergoing natural FET cycles lead to improved outcomes (Groenewoud *et al*., 2013; Ghobara & Vandekerchove, 2008). Given the dramatic rise in FET cycles over recent years, it is imperative to investigate whether the varying doses and routes of estrogen administration can affect endometrial receptivity and hormonal profile.

Estradiol can be administered via many routes- oral, transdermal, intramuscular and vaginal. To circumvent first-pass hepatic metabolism, E2 can also be administered via various parenteral routes: transdermal, intramuscular, or vaginal. Because of differences in E1/E2 ratios, transdermal E2 has been suggested to be superior to oral estrogen for inducing endometrial receptivity, and it is certainly an excellent alternative in those cases in which oral E2 does not provide adequate endometrial proliferation; even though the route of estrogen administration has not been shown to influence pregnancy rates (Krasnow *et al*., 1996; Rosenwaks *et al*., 1988; Schmidt *et al*., 1989; Aharon *et al*., 2019).

In a natural menstrual cycle, the follicular phase is about 14 days, but may vary quite widely, and still be followed by a normal luteal phase and normal endometrial receptivity. Some authors reported that pregnancy rate per cycle was comparable when estrogen was administered for 6 to 11 days before progesterone initiation but dropped significantly thereafter. Then, in this study, we used the artificial protocols with duration of 10 to 14 days of E2 stimulation (Michalas *et al*., 1996). Several studies show that the dose of estrogen in the endometrial preparation varies between 4 and 6 mg per day, with no difference in the pregnancy rate. Based on these results, our endometrial preparation protocols, regardless of the mode of administration, were performed with a daily dose of 6mg (Sekhon *et al*., 2019).

The measurement of endometrial thickness on transvaginal ultrasound alone may have sufficient predictive value to be used in lieu of an endometrial biopsy. A preovulatory endometrial thickness of 7mm or more is considered the cutoff for endometrial receptivity, below which many physicians would cancel an embryo transfer (Hofmann *et al*., 1996; Shapiro *et al*., 1993; Isaacs *et al*., 1996; Weissman *et al*., 1999; Wu *et al*., 2014). A recent study demonstrated that both clinical pregnancy and live birth rates decreased significantly for each millimeter increment below 8mm in the preovulatory phase in more than 24.000 fresh IVF-ET cycles for each millimeter increment, and below 7mm in more than 20.000 FET cycles (Liu *et al*., 2018). The endometrium thickness threshold of 7mm is used clinically in our practice. By excluding patients with <7mm endometrial thickness and no uterine alteration, we theoretically excluded uterine factor cases. To try to reduce any measurement bias, two of the authors independently measured the endometrial thickness from recorded images while blinded to the pregnancy outcome.

Luteal phase deficiency is a common result of assisted reproductive technologies (ART), and it is characterized by inadequate or inappropriate P production. The provision of exogenous progestogens to supplement endogenous P production has become a routine component of ART. Progestogen supplementation is beneficial for clinical pregnancy rates, ongoing pregnancy, and live birth versus placebo or no treatment in a Cochrane review of 875 women across eight randomized controlled trials (Wang *et al*., 2017; Basile & Garcia-Velasco, 2016; van der Linden *et al*., 2015).

About P administration routes, intramuscular provides the highest levels of circulating serum P, yet it requires a painful injection. Vaginal P is readily absorbed by the vaginal epithelium and there appears to be selective uptake of vaginally administered steroids by the endometrium. Vaginal progestogen preparations may be preferred by patients to IM preparation; therefore, we selected this administration route (Cicinelli & de Ziegler, 1999; Cicinelli *et al*., 2000; Yanushpolsky *et al*., 2010; Propst *et al*., 2001). Although various P regimens considered for endometrial preparation have been widely reported to have comparable pregnancy rates (Berger & Phillips, 2012; Shapiro *et al*., 2014; Vaisbuch *et al*., 2014).

Advances in vitrification have transformed embryo cryopreservation into a highly efficient, reliable laboratory procedure. With vitrification technology, we could do various forms of assisted reproduction technology (ART), such as preimplantation genetic testing (PGT), fertility preservation, single embryo transfer and freeze-only cycles. Due to this greater demand in the last years for knowledge on endometrial preparation, we then carried out this study and concluded that regardless of the estrogen administration mode, the hormonal profile and the pregnancy rate are the same with oral estrogen, the patch and the gel. Well designed, prospective, clinical trials are needed to confirm these results.

## CONCLUSION

Successful human reproduction depends upon a receptive endometrium. Receptivity to implantation can be induced with exogenously administered E2 and P, utilizing a variety of regimens, doses, durations and routes of administration. Adequate E2 priming is necessary for both endometrial proliferation and the induction of P receptors. For E2 administration, the simplest regimen may be the best approach. Therefore, in FET cycles, there were no differences in pregnancy rates and hormonal profile concerning E2 priming with oral (Primogyna), transdermal patches (Estradot), or transdermal gel (Oestrogel Pump).
